# Molecular characterization of DDT resistance in *Anopheles gambiae* from Benin

**DOI:** 10.1186/1756-3305-7-409

**Published:** 2014-08-29

**Authors:** Innocent Djègbè, Fiacre R Agossa, Christopher M Jones, Rodolphe Poupardin, Sylvie Cornelie, Martin Akogbéto, Hilary Ranson, Vincent Corbel

**Affiliations:** Ecole Normale Supérieure de Natitingou, Université de Parakou, BP 123 Parakou, Benin; Institut de recherche pour le développement (IRD), Maladies Infectieuses et Vecteurs, Ecologie, Génétique, Evolution et Contrôle (MIVEGEC), UM1-CNRS 5290-IRD 224, 01 BP 4414 RP Cotonou, Bénin; Centre de Recherche Entomologique de Cotonou (CREC), 06 BP 2604, Cotonou, Bénin; Département de Zoologie, Faculté des Sciences et Techniques (FAST), Université d’Abomey Calavi (UAC), BP 526 Cotonou, Bénin; Insect Migration & Spatial Ecology; Group AgroEcology Rothamsted, Research Harpenden, Hertfordshire, AL5 2JQ UK; Vector Group, Liverpool School of Tropical Medicine (LSTM), Liverpool, L3 5QA UK; Department of Entomology, Faculty of Agriculture, Kasetsart University, Bangkok, 10900 Thailand

**Keywords:** *An. gambiae*, Insecticide resistance, qPCR, Kdr mutation, Vector control, Metabolic enzymes

## Abstract

**Background:**

Insecticide resistance in the mosquito vector is the one of the main obstacles against effective malaria control. In order to implement insecticide resistance management strategies, it is important to understand the genetic factors involved. In this context, we investigated the molecular basis of DDT resistance in the main malaria vector from Benin.

**Methods:**

*Anopheles gambiae* mosquitoes were collected from four sites across Benin and identified to species/molecular form. Mosquitoes from Cotonou (M-form), Tori-Bossito (S-form) and Bohicon (S-form) were exposed to DDT 4% at a range of exposure times (30 min to 300 min). Another batch of mosquitoes from Cotonou and Malanville were exposed to DDT for 1 hour and the survivors 48 hours post exposure were used to quantify metabolic gene expression. Quantitative PCR assays were used to quantify mRNA levels of metabolic enzymes: *GSTE2*, *GSTD3*, *CYP6P3* and *CYP6M2*. Expression (fold-change) was calculated using the ∆∆Ct method and compared to susceptible strains. Detection of target-site mutations (*L1014F*, *L1014S* and *N1575Y*) was performed using allelic discrimination TaqMan assays.

**Results:**

DDT resistance was extremely high in all populations, regardless of molecular form, with no observed mortality after 300 min exposure. In both DDT-survivors and non-exposed mosquitoes, *GSTE2* and *GSTD3* were over-expressed in the M form at 4.4-fold and 3.5-fold in Cotonou and 1.5-fold and 2.5-fold in Malanville respectively, when compared to the susceptible strain. The *CYP6M2* and *CYP6P3* were over-expressed at 4.6-fold and 3.8-fold in Cotonou and 1.2-fold and 2.5-fold in Malanville respectively. In contrast, no differences in *GSTE2* and *CYP6M2* were observed between S form mosquitoes from Tori-Bossito and Bohicon compared to susceptible strain. The *1014 F* allele was fixed in the S-form and at high frequency in the M-form (0.7-0.914). The frequency of *1575Y* allele was 0.29-0.36 in the S-form and nil in the M-form. The *1014S* allele was detected in the S form of *An. gambiae* in a *1014 F/1014S* heterozygous specimen.

**Conclusion:**

Our results show that the *kdr 1014 F*, *1014S* and *1575Y* alleles are widespread in Benin and the expression of two candidate metabolic markers (*GSTE2* and *CYP6M2*) are over-expressed specifically in the M-form.

## Background

The development of insecticide resistance in *anopheles* mosquitoes is a major threat for malaria vector control. In *Anopheles gambiae* mosquitoes, the main malaria vector in Africa, two main mechanisms of resistance have been widely studied: target site modifications and insecticide detoxification known as metabolic resistance [1].

For the former, two alternative substitutions occur at position 1014 in the voltage gated sodium channel (VGSC) of *An. gambiae*: leucine to phenylalanine (*L1014F*) and leucine to serine (*L1014S*). The distribution of these two alleles is currently expanding in the M and S molecular forms of *An. gambiae* as well as in *An. arabiensis* [2]. Furthermore, the frequency of these alleles is rising in many areas of Africa associated with selective sweeps [3]. These mutations are associated with cross resistance to DDT and pyrethroids [4]. Clear association between DDT or pyrethroids resistance and the presence of kdr mutations has been shown in several studies [5]. Recently, the emergence of a new mutation *N1575Y*, within the linker between domains III-IV of the VGSC was found in *An. gambiae. N1575Y* occurs inextricably with *L1014F* on the same haplotypic background and evidence suggests that a secondary selective sweep associated with resistance to pyrethroids/DDT is occurring throughout West Africa [6].

Metabolic resistance results from increased detoxification processes by gene amplification and/or expression [1, 7–9]. The over-expression of P450 monooxygenases has been described from several pyrethroid-resistant populations of *An. gambiae* [8, 10–13] and *An. arabiensis* [14] *.* In this enzyme family, *CYP6M2* is a promising genetic marker for pyrethroid/DDT resistance as it has been demonstrated to metabolize both insecticide classes [15]. A second family of metabolic enzymes, glutathione-S-transferases (*GSTs*), is thought to play a significant role in DDT and pyrethroid resistance in *An. gambiae* [8, 16].

While the epidemiological consequences of pyrethroids resistance have yet to be established, the rapid evolution of insecticide resistant alleles over the past decade is a real cause for concern for vector control [17]. Monitoring these markers of pyrethroids resistance has significant advantages for insecticide resistance management. In Benin, entomological surveys carried out since 2007 have implicated the involvement of *GSTs*, *P450s* and esterases in insecticide resistance in *Anopheles* mosquitoes [8, 18]. The kdr mutations coupled with metabolic resistance was reported in several *An. gambiae* populations with variation described between species, sites and collection periods [8]. This situation is worrying since it can seriously threaten the efficiency of insecticide treated nets and insecticide residual spray as recently reported in Benin [19–21]. A better understanding of the genetic and evolutionary processes involved in insecticide resistance is essential to design insecticide resistance management strategies. In this study, we investigated the distribution of the *kdr* alleles and the gene expression of four candidate metabolisers of pyrethroids/DDT (*CYP6M2, CYP6P3, GSTD3* and *GSTE2*) in *An. gambiae* throughout Benin.

## Methods

### Mosquito sampling

From December 2010 to December 2011, larvae of *An. gambiae* mosquitoes were collected in four different sites in Benin (Cotonou, Tori-Bossito, Bohicon and Malanville) in the framework of the WHO/TDR network project [18]. All larvae were brought back to laboratory of the Centre de Recherche Entomologique de Cotonou (CREC) for rearing. Emerging adult female mosquitoes (F_0_) were used for insecticide susceptibility tests and molecular assays.

The IRD (Institut de Recherche pour le Développement) Ethics Committee and the National Research Ethics Committee of Benin approved the study (CNPERS, reference number IRB00006860).

### Insecticide susceptibility tests

Insecticide susceptibility tests were carried out on 2–5 days old female mosquitoes [22]. Samples collected in Cotonou and Malanville in December 2010 were exposed to DDT 4% for 1 hour and survivors of 48 hours after DDT exposure were stored in RNA later (SIGMA). In December 2011, WHO cylinder kits were used to expose mosquitoes (from Cotonou, Tori-Bossito and Bohicon) to increasing exposure times with the intention of generating time-response curves. Batches of 20–25 mosquitoes were exposed to test papers impregnated with DDT 4% at the following exposure times; 30 min, 45 min, 60 min, 90 min, 120 min, 150 min, 240 min and 300 min. Non-impregnated control papers were used throughout all experiments. Survivors and non-exposed mosquitoes were also stored in RNA later (SIGMA) and kept at −20°C for DNA and RNA analysis.

### Species identification and *kdr*genotyping

DNA was extracted from control and alive mosquitoes following insecticide exposure using the LIVAK buffer method [23]. Specimens were identified to species and molecular form by the SINE-PCR protocol [2]. *L1014F*, *L1014S* and *N1575Y* were screened using TaqMan assays as previously described [6, 24]. Forward and reverse primers and three minor groove binding (MGB) probes (Applied Biosystems) were designed using the Primer Express™ Software Version 2.0. Primers *kdr*-Forward (5' CATTTTTCTTGGCCACTGTAGTGAT-3'), and *kdr*-Reverse (5'-CGATCTTGGTCCATGTTAATTTGCA-3') were standard oligonucleotides with no modification. The probe WT (5'-CTTACGACTAAATTTC-3') was labelled with VIC at the 5' end for the detection of the wild type allele, the probes *kdr*W (5'-ACGACAAAATTTC-3') and *kdr*E (5'-ACGACTGAATTTC- 3') were labelled with 6-FAM for detection of the *kdr*-w and *kdr*-e alleles respectively. For the N1575Y, the primers F3’TGGATCGCTAGAAATGTTCATGACA-5’ R3’CGAGGAATTGCCTTTAGAGGTTTCT-5’were used [6].

### mRNA expression of candidate metabolic genes

#### RNA extraction and cDNA synthesis

Mosquitoes which survived DDT exposure (300 min) were used for the qPCR assays. Total RNA was extracted from batches of five mosquitoes (stored in RNA later) for each replicate by using PicoPure™ RNA kit isolation (Arcturus) according to the manufacturer’s instructions. RNA was treated using the RNA-Free DNAse set (Qiagen) to remove any contaminating genomic DNA. The concentration and the quality of the total RNA were assessed using a Nanodrop spectrophotometer (Nanodrop Technologies, UK). SuperSript™ III Reverse Transcriptase was used to synthesize first strand cDNA.

### Reverse-transcription quantitative PCR (RT-qPCR)

The relative gene expression of *CYP6M2, CYP6P3, GSTD3* and *GSTE2* was analyzed by quantitative PCR (qPCR). Actin-5C (AGAP000651) and ribosomal protein S7 (AGAP010592) were used as endogenous control genes to account for any differences in template input. Three biological replicates were run for each sample on a plate. A TaqMan gene expression assay was used for *GSTE2* whereas SYBR Green was used for *CYP6M2*, *CYP6P3* and *GSTD3*. Primers for qPCR were designed using NCBI primer BLAST (http://www.ncbi.nlm.nih.gov/tools/primer-blast/) by using Xm codes from Vector Base. The Table 1 shows the primers used for qPCR.

**Table 1:** **Primers used in quantitative real-times PCR (qPCR) (F = Forward; R = Reverse)**

Primers	Primer sequences	References
CYP6P3	F: 5’-GTGATTGACGAAACCCTTCGGAAGT-3’	[25]
	R: 5’-GCACCAGTGTTCGCTTCGGGA-3’	
CYP6M2	F: 5’-TACGATGACAACAAGGGCAAG- 3’	
	R: 5’- GCGATCGTGGAAGTACTGG-3’	
GSTD3		
	F: 5’-CTAAGCTTAATCCGCAACATACCA-3	
	R: 5’-GTGTCATCCTTGCCGTACAC-3’	
GSTE2	F: 5’-GCCGGAATTTGTGAAGCTAAACCCG-3’	
	R: 5’-TGCTTGACGGGGTCTTTCGGAT-3’	
S7	F: 5’- AGAACCAGCAGACCACCATC-3’	[14]
	R: 5’- GCTGCAAACTTCGGCTATTC-3’	
Actin	F: 5’-ACATCGCCGAAGATCGCCCA-3’	
	R: 5’-AGAGGGATTAAGTTGCAGCACTCG-3’	

The expression of *CYP6M2* and *GSTE2* was determined in non-exposed (control batches) and exposed (DDT 300 minutes) mosquitoes. The Kisumu strain (susceptible; S-form) and N’Gousso (susceptible; M-form) were used as reference laboratory strains and not selected with insecticide. Real-time PCR reactions were run on the Agilent MxP3005P (Agilent Technologies). For each target gene, standard curves were generated using a five times serially diluted cDNA samples to assess the PCR efficiency and the dynamic range of cDNA. The PCR efficiencies of each gene fell ±10% of 100% and all had single melting curve peaks indicating specificity of the assay. The cDNA were diluted 5-fold in as this was the concentration that fitted within the dynamic range of each qPCR and stored at −20°C.

### Data analysis

The mortality rates were classified in accordance with the recommended criteria by WHO [22]. The resistance status was determined based on the following criteria:

- Mortality > 97%: susceptible Anopheles population.
  - Mortality 80 – 97%: suspected resistance in the Anopheles population.
  - Mortality < 80%: resistant Anopheles population

Our hypothesis according to previous findings with the metabolic candidate genes was that gene expression is higher in resistant-field mosquitoes than the laboratory susceptible strain. Therefore, the relative expression (linear fold-changes) of *CYP6M2, CYP6P3*, *GSTD3* and *GSTE2* were calculated according to the ΔΔCt method described by Schmittgen, and Livak [26] using laboratory strains as calibrator samples. Strains were only compared belonging to the same molecular form (e.g. Cotonou/Malanville versus N’gousso (M-form) and Tori-Bossito/Bohicon versus Kisumu (S-form). PCR efficiencies were incorporated into the calculations. Basic data analysis (regression and t-tests were performed in Excel with p < 0.05 used to assess significant difference between treatments for the t-tests).

## Results

### DDT resistance

In 2010, bioassays showed strong resistance to DDT in *An. gambiae* in two parts of the country (1% and 6% mortalities in Malanville and Cotonou respectively) [18]. In 2011, we observed no mortality in mosquitoes exposed to 4% DDT regardless of the exposure time (from 30 to 300 minutes), indicating that all mosquito populations were strongly resistant to this insecticide.

### Differential expression of metabolic genes in *An. gambiae*

The pre-dominant molecular form in Cotonou (M-form), Malanville (M-form), Bohicon (S-form) and Tori-Bossito (S-form) was exclusively used for qPCR analysis to avoid any bias in expression from mixed molecular forms.

**Figure 1:**
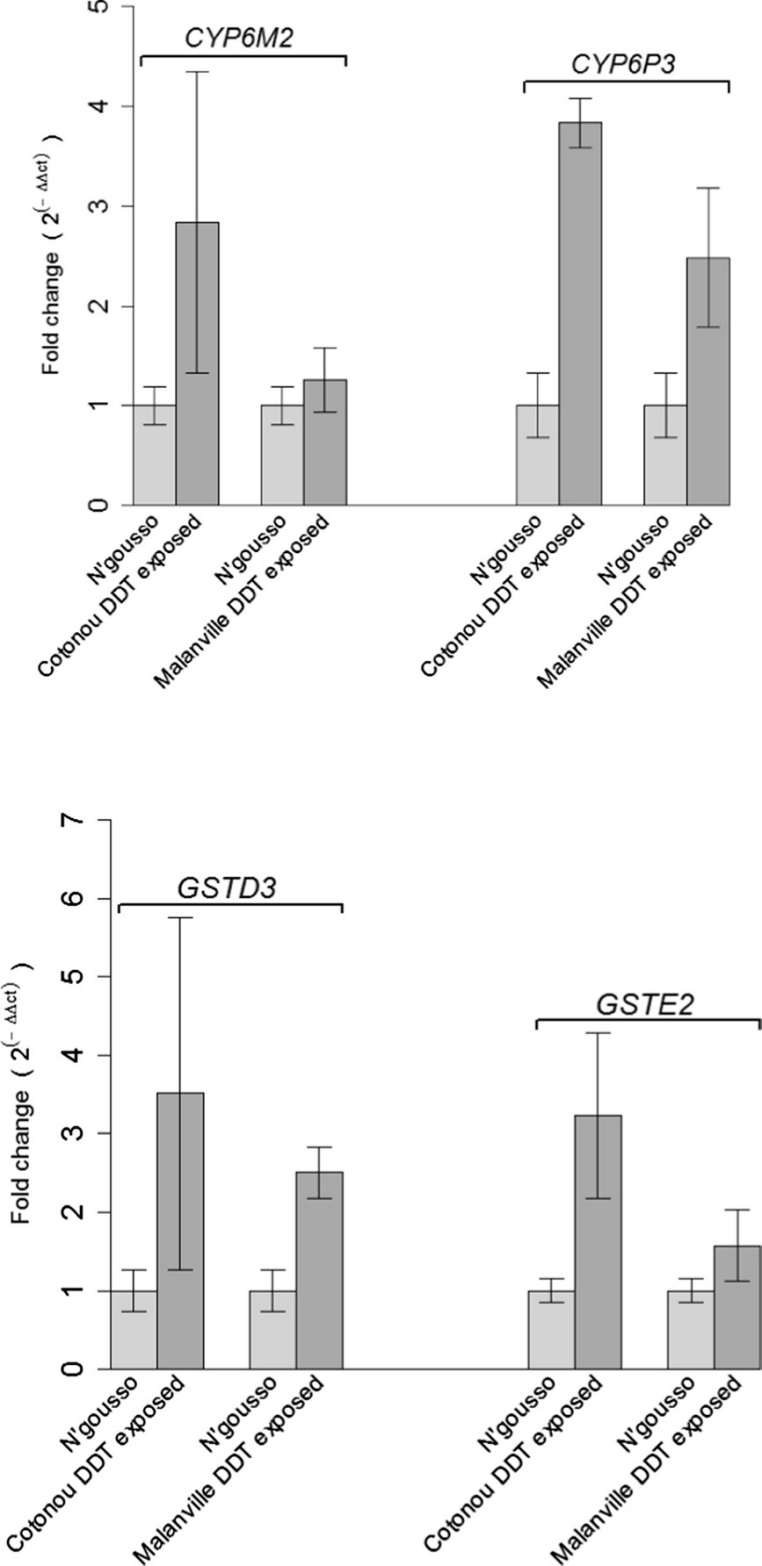
**The** ***CYP6M2*** **,** ***CYP6P3*** **,** ***GSTE2*** **and** ***GSTD3*** **expression in Cotonou and Malanville after 48 hours DDT-exposure compared to those of. N’Gousso (M-form laboratory mosquito) Error bars are 95% confidence interval.**

Two experiments were performed to analyse the gene expression of metabolic candidates between the different strains from Benin. In the first experiment, M-form mosquitoes from Malanville and Cotonou collected in 2010 were exposed to DDT for one hour and the gene expression of *GSTE2*, *GSTD3*, *CYP6P3* and *CYP6M2* compared to the laboratory strain N’Gousso. All genes were up-regulated in DDT-exposed mosquitoes from Cotonou (between 2.8 and 3.8-fold (2^-ΔΔCt^)) (p < 0.05) whereas only *CYP6P3* (2.4-fold) (p = 0.026) and *GSTD3* (2.5-fold) (p = 0.020) were over-expressed in Malanville (Figure 1).

**Figure 2:**
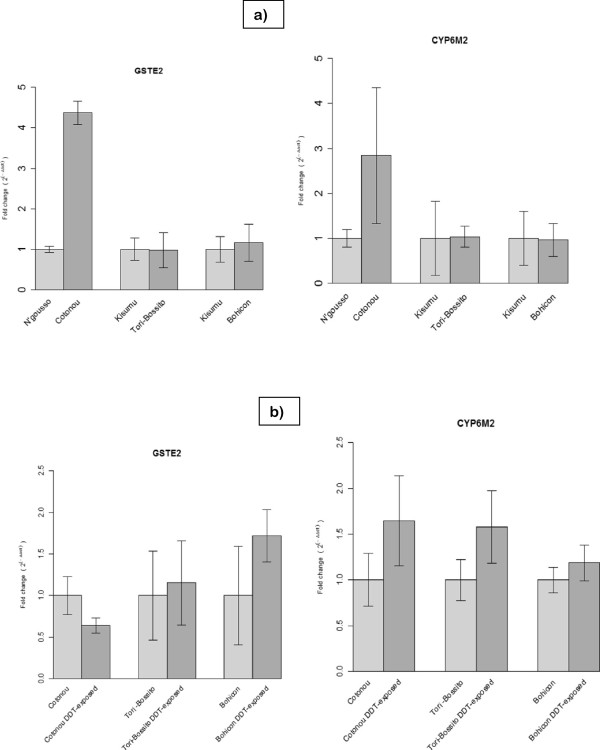
**The** ***GSTE2*** **and** ***CYP6M2*** **expression in non-exposed and DDT-exposed** ***An. gambiae s.l*** **collected in Cotonou, Tori-Bossito and Bohicon.** **a)** The *GSTE2* and *CYP6M2* expression in *An. gambiae s.l* collected in Cotonou, Tori-Bossito and Bohicon compared to those of N’Gousso (M-form laboratory mosquito) and Kisumu (S-form laboratory mosquito). Error bars are 95% confidence interval. **b)** The *GSTE2* and *CYP6M2* expression in *An. gambiae s.l* collected in Cotonou, Tori-Bossito and Bohicon after 300 min exposure to 4% DDT compared to those of non-exposed mosquitoes. Error bars are 95% confidence interval.

In the second experiment, the gene expression of *GSTE2* and *CYP6M2* was compared between collections from three sites in 2011. Expression of each gene was compared between (i) mosquitoes exposed to DDT (ii) non-exposed resistant mosquitoes and (iii) laboratory susceptible strains. There were notable differences in expression between the wild mosquitoes and the laboratory strains (Figure 2). In M-form mosquitoes from Cotonou *GSTE2* and *CYP6M2* were up-regulated to 4.37 (p = 0.0013) and 2.23-fold (p = 0.037) compared to N’Gousso. In contrast, these genes did not show over-expression in the S-form in Tori-Bossito and Bohicon compared to Kisumu (p > 0.05). No significant differences in *GSTE2* and *CYP6M2* expression between DDT-exposed and non-exposed mosquitoes observed in any populations (p > 0.05).

### Target-site mutations in *An. gambiae*in Benin

The *N1575Y* and *L1014F* mutations were identified in both non-exposed M- and S- form of *An. gambiae* in Benin. The *L1014F* kdr allelic frequency was almost fixed in the S form (0.932-1.00), which predominates in the south of the country. The frequency of *L1014F* ranged between 0.67 and 0.91 in the M-forms in the south (Cotonou, Tori Bossito and Bohicon) and was 0.81 (0.691-0.891) in the north (Malanville).

In 2010, we found the *1575Y* allele in both molecular forms of *An. gambiae*. In the M-form, the frequency of this allele was much higher in the northern site Malanville; 0.321 (95% CI 0.214-0.452) than in the southern site of Cotonou 0.019 (95% CI 0.003-0.098) (p = 0.00). In 2011 we did not detect the *1575Y* allele in Cotonou whereas the frequencies of *1575Y* were 0.291 (95% CI 0.223-0.368) in Bohicon and 0.36 (95% CI 0.267-0.466) in Tori Bossito (see Table 2). There was no significant difference in the *1575Y* frequency between years (p = 0.071).

**Table 2:** ***Kdr*** **allelic frequencies in** ***An. gambiae*** **in each site per collection period**

M-form						
2010	*f* 1014 L (95% C.I.)	*f* 1014 F (95% C.I.)	*N* (alleles)	*f* 1575 N (95% C.I.)	*f* 1575Y (95% C.I.)	*N* (alleles)
Cotonou	0.10 (0.048-0.208)	0.90 (0.792-0.952)	58	0.98 (0.902-0.997)	0.02 (0.003-0.098)	54
Malanville	0.19 (0.109-0.309)	0.81 (0.691-0.891)	58	0.68 (0.548-0.786)	0.32 (0.21-0.45)	56
Tori Bossito	0.33 (0.138-0.609)	0.67 (0.391-0.862)	12	1	0	12
Bohicon	0.12 (0.022-0.471)	0.88 (0.529-0.978)	8	1	0	8
2011	*f* 1014 L (95% C.I.)	*f* 1014 F (95% C.I.)	*N* (alleles)	*f* 1575 N (95% C.I.)	*f* 1575Y (95% C.I.)	*N* (alleles)
Cotonou	0.09 (0.046-0.156)	0.91 (0.84-0.95)	104	1	0	104
Tori Bossito	0.30 (0.10-0.60)	0.70 (0.39-0.89)	10	1	0	10
**S-form**						
2010	*f* 1014 L (95% C.I.)	*f* 1014 F (95% C.I.)	*N* (alleles)	*f* 1575 N (95% C.I.)	*f* 1575Y (95% C.I.)	*N* (alleles)
Bohicon	0.05 (0.014-0.165)	0.95 (0.835-0.986)	40	0.80 (0.652-0.895)	0.20 (0.10-0.34)	40
Tori Bossito	0.068 (0.024-0.182)	0.932 (0.818-0.977)	44	0.68 (0.534-0.8)	0.32 (0.20-0.46)	44
2011	*f* 1014 L (95% C.I.)	*f* 1014 F (95% C.I.)	*N* (alleles)	*f* 1575 N (95% C.I.)	*f* 1575Y (95% C.I.)	*N* (alleles)
Bohicon	0	0.99	148	0.71 (0.632-0.777)	0.29 (0.22-0.36)	148
Tori Bossito	0	1	86	0.64 (0.534-0.733)	0.36 (0.26-0.46)	86

The *1014S* allele was identified in one specimen of *An. gambiae* S-form in Bohicon in co-occurrence with the *1014 F* allele.

## Discussion

This study showed extremely high levels of DDT resistance in field populations of *An. gambiae* in Benin. This resistance profile is likely to be due to a combination the high frequencies of kdr mutations (*L1014F* and *N1575Y)* and over-expression of metabolic genes, i.e. *GSTE2, GSTD3, CYP6M2* and *CYP6P3* known to be involved in DDT and/or pyrethroids resistance.

The *1014 F* kdr allelic frequency was almost fixed in the S-form and at a high frequency in the M-form. Such kdr *1014 F* frequency in *An. gambiae* has been recently reported throughout sub-Saharan Africa [18, 27, 28]. In 2011 the kdr *1575Y* allele was detected in the- S-form only and occurred solely upon a *1014 F* haplotypic background confirming the results of Jones *et al.* [6]. The prevalence of this mutation has increased in West Africa in the last years hence indicating that the *1014 F-1575Y* haplotype is under strong selection. From our data there was a slight but non-significant increase in *1575Y* in the S-form. The *1575Y* was only found at a high frequency in the M-form in the north of the country (0.321) close to the border of Burkina Faso where similar frequencies of this mutation have previously been observed [6]. We also detected *L1014S* from *An. gambiae* S-form confirming the extension of this mutation in *An. gambiae s.s.* in West Africa. Recent surveys carried in Benin and Burkina Faso detected the presence of *1014S* kdr allele in both M and S form and *An. arabiensis* [18, 29, 30].

The additive resistance of *1575Y* for permethrin and DDT in the S- and M-forms of *An. gambiae* respectively [6] and the presence of *1014S* highlights the importance of continually monitoring for these mutations as part of insecticide resistance management.

Based on previous findings that implicate metabolic candidate genes in DDT resistance we analysed the expression levels of two *P450s* and two *GSTs* in DDT resistant mosquitoes in Benin.

The over-expression of *CYP6M2* and *CYP6P3* has previously been associated with pyrethroid and DDT resistance in *An. gambiae* and are known metabolizers of both types I and type II of pyrethroids and DDT [11, 15]. The specific up-regulation of these two genes in the M-form from Cotonou agrees with previous findings in Benin and Ghana [7, 15]. Increased *GST* activity is known to confer DDT resistance in mosquitoes [31, 32] by catalyzing the removal of a chlorine group from the insecticide. In this study, *GSTE*2 and *GSTD3* were up-regulated in M-form mosquitoes. Delta class GSTs have been implicated in insecticide resistance [33] but their role has previously thought to be relatively minor compared with those from the epsilon class. *GSTE2* has been strongly associated with DDT and pyrethroid resistance in *An. gambiae* mosquitoes from Ghana [34–37] and with DDT resistance in *An. funestus* from Benin [38]. In this latter country, a single amino acid change (L119F) in an up-regulated glutathione S-transferase gene, GSTe2, in *Anopheles funestus* showed to confer high levels of metabolic resistance to DDT hence representing a promising marker to track the evolution of DDT and pyrethroid resistance in malaria vectors in West Africa [39, 40]. *GSTD3* was up-regulated in DDT-resistant *An. arabiensis* from an urban site in Burkina Faso [30]. Further validation of the role of *GSTD3* in DDT resistance is required.

In this paper, significant difference in gene expression between molecular forms and the laboratory strains was reported. No differential expression of *CYP6M2* and *GSTE2* was observed in the S-form from Bohicon and Tori-Bossito compared to the Kisumu strain whereas mosquitoes belonging to the M-form showed higher expression levels compared to N’Gousso. The difference of expression of these metabolic genes may be due to the differences of selection pressure induced by various xenobiotics in larval breeding sites. Indeed, general ecological differences have been documented between the M- and S-forms [41–43]. The M-form preferentially breeds in permanent polluted freshwater collections mainly resulting from human activity (e.g., agriculture and urbanization), whereas the S form thrives in temporary non-polluted breeding sites (e.g., rain-filled puddles, road ruts, and quarries) [42]. In the present study, the over-expression observed in M-form, may reflect the influence of a range of xenobiotics on selecting for resistance in mosquitoes [7, 44]. Whilst the impact of agricultural and public health use of insecticides has been widely linked to selection for resistance in malaria vector, recent evidence has also implicated other xenobiotics such as petroleum oils, heavy metals etc. [44–47]. We cannot exclude the possibility that besides these four metabolic genes, other enzymes and genetic mechanisms could be contributing to the phenotype as suggested from previous microarray studies [15, 37].

In the presence of xenobiotics, metabolic resistance can be related to constitutive or induced detoxification process or both [48]. Here we analyzed the induction effect of *GSTE2* and *CYP6M2* in *An. gambiae* mosquitoes after 300 minutes exposure to DDT. Results showed that mosquito exposure to DDT did not induce over-expression of *GSTE2* and *CYP6M2*, suggesting that two genes are constitutively over-expressed in resistant mosquitoes.

The evidence that malaria vectors exhibit multiple insecticide resistance mechanisms is worrying for malaria prevention in Africa [4]. In Benin, reduced efficacy of LLIN and IRS has been shown in areas where malaria vectors exhibits high *1014 F* frequency [21, 49]. There is an urgent need to implement routine insecticide resistance monitoring through all Malaria Control Programmes relying on DDT-based treatments. Monitoring the frequency and distribution of the genes contributing towards the resistance phenotype should play a role in insecticide resistance management. Quantifying the expression of the candidate genes analysed here using robust RT-qPCR assays in populations of resistant-mosquitoes throughout Benin could help this process.

## Conclusion

The spread of multi-resistance in wild populations of *An. gambiae* is worrying as it could threaten the effectiveness of malaria vector control strategies based on the use of chemicals.

## Abbreviations

ITNs: Insecticide-treated nets
LLINs: Long-lasting insecticide treated nets
IRS: Indoor residual spraying
DDT: Dichlorodiphenyltrichloroethane
GSTs: Glutathione S-transferases
P450: Cytochrome P450 monooxygenase enzymes
RNA: Ribonucleic acid
qPCR: Quantitative Polymerase Chain Reaction
RT-qPCR: Reverse transcription and quantitative polymerase chain reaction
cDNA: Complementary Deoxyribonucleic acid
CYP: genes encoding cytochrome
WHO: World Health Organization.
